# Protective effects of fentanyl preconditioning on cardiomyocyte apoptosis induced by ischemia-reperfusion in rats

**DOI:** 10.1590/1414-431X20165286

**Published:** 2017-02-16

**Authors:** Q. Xu, Q.-G. Li, G.-R. Fan, Q.-H. Liu, F.-L. Mi, B. Liu

**Affiliations:** 1 Department of Anesthesiology, Linyi People's Hospital, Linyi, Shandong, China; 2 Department of Anesthesiology, Linyi Cancer Hospital, Linyi, Shandong, China; 3 Operation Room, Linyi People's Hospital, Linyi, Shandong, China

**Keywords:** Fentanyl, Ischemia-reperfusion, Myocardial apoptosis, Myocardial infarction, Hemodynamic parameters, B-cell lymphoma 2, Bax

## Abstract

We aimed to study the effect of fentanyl (Fen) preconditioning on cardiomyocyte apoptosis induced by ischemia-reperfusion (I/R) in rats. A total of 120 Sprague Dawley male rats (age: 3 months) were randomly divided into: sham operation group (S group), I/R group, normal saline I/R group (NS group), and fentanyl low, middle, and high dose groups (Fen1: 2 μg/kg; Fen2: 4 μg/kg; Fen3: 6 μg/kg). Heart rate (HR), mean arterial pressure (MAP), left ventricular developed pressure (LVDP), ±dp/dtmax, malondialdehyde (MDA), superoxide dismutase (SOD) activity, creatine phosphokinase-MB (CK-MB), and cardiac troponin-I (cTnI) were measured. Myocardial ischemic (MI) area, total apoptotic myocardial cells, and protein and mRNA expressions of B-cell lymphoma 2 (Bcl-2) and Bax were detected. HR and MAP were higher, while LVDP and ±dp/dtmax were close to the base value in the Fen groups compared to those in the I/R group. Decreased MDA concentration and CK-MB value and increased SOD activity were found in the Fen groups compared to the I/R group, while cTnI concentration was significantly lower in the Fen1 and Fen2 groups (all P*<*0.05). Myocardial damage was less in the Fen groups compared to the I/R group and the MI areas and apoptotic indexes were significantly lower in the Fen1 and Fen2 groups (all P*<*0.05). Furthermore, significantly increased protein and mRNA expressions of Bcl-2, and decreased protein and mRNA expressions of Bax were found in the Fen groups compared to the I/R group (all P<0.05). Fentanyl preconditioning may suppress cardiomyocyte apoptosis induced by I/R in rats by regulating Bcl-2 and Bax.

## Introduction

Myocardial infarction (MI), a common presentation of coronary artery disease, is characterized, in part, by myocardial necrosis induced by persistent and severe myocardial ischemia (1). Ischemic heart disease is the primary cause of mortality and morbidity all over the world (2). Rapid reperfusion is critical in the treatment of myocardial ischemic incidents, and blood flow restoration is necessary for the salvage of endangered myocardium after MI (3,4). Ischemic/reperfusion (I/R) injury reflects the rapid increase of tissue damage after a certain period of reperfusion to ischemic tissue (4,5). Recently, necrosis and apoptosis were suggested to be the two forms of damage resulting from reperfusion injury, and have been considered to be a persistent problem in the treatment of myocardial ischemia (6,7). Previous studies have shown that reperfusion can lead to energy metabolism disorder of myocardial cells, change of myocardial ultrastructure and apoptosis of the myocardial cell (8,9). Ischemic preconditioning (IPC) strategies were used to protect tissues against I/R injury; furthermore, opioid drugs have been confirmed to have a protective effect against I/R injury (10,11).

Fentanyl, known as an opioid analgesic synthesized by phenylpiperidine-derivative, is structurally correlated to meperidine and works primarily at the μ-receptor, and is commonly used in neurosurgery as the pre-induction adjunct (12,13). Several studies have shown that fentanyl is absorbed rapidly and reaches the maximum serum level in around 2 minutes with few cardiovascular effects without histamine release. It is suggested to be 75–100 times more effective than morphine (14,15). However, fentanyl has several side effects such as nausea and vomiting, bradycardia and respiratory depression, which are often in a dose-related manner (15). Importantly, it has also been reported that fentanyl may protect the heart from post-ischemic injury (16).

However, the underlying mechanisms of fentanyl and its association with the cardiomyocyte apoptosis of I/R injury remains unknown. In this regard, we established a rat model to observe the effects of fentanyl preconditioning on myocardial cell apoptosis induced by I/R injury.

## Material and Methods

### Animals and grouping

A total of 120 Sprague Dawley male rats (body weight: 200–250 g; age: 3 months) were provided by the Linyi People's Hospital. The test rats were kept in an adequate experimental environment (room temperature: 25°C; humidity: 40–60%; lighting: 12 h per day) for 7 days before the experiment, and fasted for 12 hours before the establishment of myocardial I/R injury model. The rats were randomly divided into six groups (n=20 for each group): sham operation group (S group), I/R group, normal saline I/R group (NS group), fentanyl low dose group (Fen1 group, 2 μg/kg), fentanyl middle dose group (Fen2 group, 4 μg/kg), and fentanyl high dose group (Fen3 group, 6 μg/kg). The experiments were carried out in strict accordance with the guidelines of the National Institutes of Health for the use of laboratory animals (17), and procedures were approved by the Linyi People's Hospital. All traumatic procedures were performed under anesthesia, and all efforts were made to minimize suffering.

### Myocardial I/R injury model establishment

The rats were fasted for 12 h before the establishment of the myocardial I/R injury model and had free access to water. Preoperative electrocardiogram was conducted on rats to obtain a lead electrocardiograph (ECG), and rats with abnormal ECG were excluded. The included rats were anesthetized with 1.5 mL/kg sodium pentobarbital (30 g/L) by intraperitoneal injection, fixed, subjected to tracheal cannula, and connected to an ALC-V model animal ventilator with a breathing frequency of 50–60 bpm. ECGs of the rats were continuously recorded. Then, about 2 cm of chest skin was longitudinally cut along the left midclavicular line, the muscles of sternum were longitudinally clamped by a hemostatic clamp several times, and then the muscles were cut with scissors. A small opening was bluntly separated at the intercostal of the 2nd–3rd ribs in the left side near the sternum, approximately horizontal to the underarm location. The chest was carefully cut open and the pericardium was cut to fully expose the rat heart, and the left atrial appendage and the pulmonary cone were found. A 5th size needle and thread was inserted vertically at the lower edge of the left atrial appendage, and was withdrawn at the junction of the rat left atrial appendage and the pulmonary cone. Left anterior descending artery (LAD) was ligated. ST segment elevation indicated the successful establishment of the MI model. After the ligation of the left ventricular apex and inferior wall, decreased blood pressure and ECG changes were found in the rats. After reperfusion was conducted, local red color was shown and the ST-segment depression was presented on ECG. In the S group the chest was opened and threaded without ligation. In the I/R group the, chest was opened, the left ventricular branch was threaded, ligated for 30 min, and then subjected to reperfusion for 120 min. In the NS group, before ischemia, normal saline was intravenously infused for 5 min, followed by a 5 min interval (this was repeated 3 times), and then ligation was carried out for 30 min and reperfusion for 120 min. In the Fen 1, 2, and 3 groups: before ischemia, fentanyl (2, 4, and 6 μg/kg, respectively) was intravenously infused for 5 min, followed by a 5 min interval, (this was repeated 3 times), and ligation was conducted for 30 min and reperfusion for 120 min.

### Hemodynamic parameter detection

For the experimental rats, needle tubes were preserved in the carotid artery and connected to a pressure transducer. The specific operation methods were as follows: on the basis of the tracheal cannula, the right carotid artery was separated. A 1.0-silk thread was used to ligate the artery at the distal end and a 45-degree tilt incision was made on the artery wall about 1 cm below the ligation thread using ophthalmic scissors. The artery was inserted with a catheter and connected to a pressure transducer to observe arterial pressure pattern changes. Hemodynamic parameters such as heart rate (HR), mean arterial pressure (MAP), left ventricular developed pressure (LVDP), left ventricular pressure change rate (±dp/dtmax) of each group at a basic state (T0), immediately before ischemia (T1), ischemia for 30 min (T2), reperfusion for 30 min (T3), reperfusion for 60 min (T4), and reperfusion for 120 min (T5) were recorded.

### Biochemical parameter detection

Right carotid artery blood samples (2 mL) of rats in each group were taken at T0 and T5, left standing for 2 h, and centrifuged at 680 *g* at room temperature for 10 min. The upper layer of serum was stored at –80°C. Thiobarbituric acid reaction method was used for the determination of malondialdehyde (MDA), xanthine oxidase for super oxide dismutase (SOD) and immunosuppressive method for creatine phosphokinase-MB (CK-MB). The operations were conducted according to the MDA, SOD and CK-MB (purchased from Nanjing Jiancheng Bio Co., Ltd., Nanjing, China) kits’ instructions. Plasma concentration of cardiac troponin-I (cTnI) was determined at T5. In details, blood samples (2 mL) were placed in a clean test tube with anticoagulant. The operation steps were in strict accordance with the kit (purchased from Shanghai Seebiotech Biological Technology Co., Ltd., Shanghai, China) instructions. Colloidal gold immune chromatography was applied and automatic immunoassay analyzer was used to conduct measurement.

### Histological and morphological change detection

Hematoxylin-eosin staining was used to observe the histological and morphological changes. At the end of reperfusion, the hearts were removed and rinsed with iced normal saline. Atriums, right ventricles, and connective tissues were removed, the left ventricular apex was taken, and two myocardial slices were cut at a thickness of 0.2 cm. The myocardial slices were placed in a 5% formaldehyde solution for 24 h, then rinsed with phosphate buffered saline, and embedded in paraffin. The paraffin-embedded slices were cut into a thickness of 5 cm and observed after staining.

### MI area detection

Even's blue-2,3,5-triphenyl tetrazolium chloride (TTC) method was used to detect MI area. Blood was extracted at the end of reperfusion, LAD was ligated again. Evan's blue (5%, 2 mL) was injected into the tail vein. When the myocardial cells in the non-ischemic zone appeared dark blue, the hearts were rapidly taken out and weighed after being dried with filter paper. The left ventricle (LV) was separated, weighed and then frozen at -20°C for 1 h. The frozen LV was cut into 6 myocardium slices (2 mm/slice) along the long axis. The LV slices were placed in 1% TTC solution at a pH of 7.4 and then incubated in thermostating water at 37°C, followed by fixation in 10% formaldehyde for 15 min. Afterward, the slices were photographed and weighed. Due to the fact that live cells contain dehydrogenase and TTC can be reduced into a deep red color, the infarct size (IS) cannot be stained and presented gray white color. The ranges of area at risk (AAR) and IS were determined by quadrature with Image-Pro Plus 5.0 (Media Cybernetics, USA). The weight of each myocardium slice was adjusted for calculation of the total weight of the left ventricle and the results were presented as percentages. The myocardial ischemic area was calculated as AAR weight/LV weight; and the MI area was IS weight/AAR weight. IS = Σ [(A1×W1) + (A2×W2) + (A3×W3) + (A4×W4) + (A5×W5) + (A6×W6)] ×100%, AAR=Σ [(R1×W1) + (R2×W2) + (R3×W3) + (R4×W4) + (R5×5) + (R6×W6)] × 100%, where IS/AAR%=Σ infarct weight in each slice/risk area weight in each slice ×100%, where A is the area of infarct for the slice, R is the area at risk for the slice in left ventricle, W is the weight of the respective section and number 1–6 relate to the six slices (18,19).

### Myocardial cell apoptosis

The 5-cm paraffin embedded slices were placed on slides and apoptosis index (AI) was measured using the terminal deoxynucleotidyl transferase-mediated dUIP nick-end labeling (TUNEL) assay. The nuclei of TUNEL-positive cells were brown under a light microscope. Image Pro Plus 4.5 image analysis software (Media Cybernetics) was used to calculate the number of apoptotic cells. Five non-overlapping high-power fields (40×) were randomly selected, and the number of apoptotic positive cell nuclei and the number of total cell nuclei were calculated. The AI of myocardial cells was calculated as: AI = (number of myocardial apoptotic cell nuclei / total number of myocardial cell nuclei) × 100%. The mean AI was obtained.

### Detection of protein expressions

Rats were sacrificed at T5; rat hearts were removed over ice, and washed with iced normal saline. The left ventricular myocardium tissue under the ligature was cut into pieces and ground in a glass-grinding vessel with the addition of protein lysate, kept standing for 30 min. The left ventricle homogenate was centrifuged continuously at 300 *g* for 10 min at 4°C. The supernatant was extracted and again centrifuged continuously at 300 *g* at room temperature for 30 min. The supernatant was extracted, sub-packaged with a concentration of 20 microliters and stored for the next step at –80°C. For analysis, the myocardial proteins were thawed, homogenized, and subjected to rewarming. The protein supernatant was transferred to a nitrocellulose membrane by SDS-polyacrylamide electrophoresis, and sealed with 10% skim milk tris-buffered saline and Tween 20 (TBST – liquid) for 1 h. Primary antibodies (1:1000; rabbit anti-mouse B-cell lymphoma 2 (Bcl-2) and Bax; purchased from Santa Cruz, USA) were added and the whole system was incubated at room temperature for 2 h and washed with TBST solution for three times. Horseradish peroxidase-labeled secondary antibodies were added and the whole system was incubated at room temperature for 2 h, washed by TBST solution three times, and developed by Electro-Chemi-Luminescence (ECL; Amersham, USA) kit. The gel-imaging system was scanned and the gray value of each band was analyzed by the Quality One software (Bio-Rad, Inc., USA). Relative protein expression levels were calculated.

### mRNA expressions of Bcl-2 and Bax in myocardial cells

Necrotic myocardial tissues (about 100 mg) of the left ventricular anterior walls were loaded in RNA enzyme contamination-free freezing tubes and preserved in liquid nitrogen. Myocardial cell Bax and Bcl-2 mRNA expressions were measured by reverse transcriptase-polymerase chain reaction (RT-PCR). Total myocardium RNA was extracted by a Trizol kit (Invitrogen Inc., USA) and the RNA concentration was detected with a UV300 ultraviolet spectrophotometer (Visible Spectrometer Company, UK). The cDNA was then synthesized. ABI7500 fluorescence quantitative PCR instrument (Applied Biosystems Inc., USA) was used to detect mRNA expressions of Bax, Bcl-2, and internal reference β-actin. Bax upstream primer: 5′-GTTACAGGGTTTCATCCAGG-3′, downstream primer: 5′-CGTGTCCACGTCAGCAAT-3′, and an amplified length of 178 bp; Bcl-2: upstream primer: 5′-TACGAGTGGGATACTGGAGA-3′, downstream primer: 5′-TCAGGCTGGAAGGAGAAG-3′, and an amplified length of 80 bp; β-actin upstream primer: 5′-CGTGCGTGACATTAAAGAG-3′, downstream primer: 5′-TTGCCGATAGTGATGACCT-3′, and an amplified length of 132 bp. Cycle parameters: 30 cycles of 95°C for 30 s; 94°C for 30 s, 57°C for 30 s, and 72°C for 1 min, followed by a terminal extension of 72°C for 10 min.

### Statistical methods

GraphPad Prism 6 statistical software (GraphPad Software, USA) was used to analyze the data. Data are reported as averages and standard deviations. Differences between groups were analyzed using *t*-test. One-way ANOVA was used to analyze data in multiple groups. P<0.05 was considered to be significant.

## Results

### Heart function changes in each group

As shown in Table 1, there were no significant differences in HR, MAP, LVDP and ±dp/dtmax of rats in each group at T0 (all P>0.05). At other time points, HR and MAP were all decreased in the I/R, NS, Fen1, Fen2 and Fen3 groups compared to the S group (all P<0.05). In addition to the S group, the other groups also presented significantly different HR and MAP at T1, T2, T3, T4 and T5 compared to that at T0 (all P*<*0.05). LVDP and +dp/dtmax, two left ventricular systolic function indicators, were lower in the I/R group during the I/R process and in the Fen3 group at T1 (all P<0.05). In addition, in the Fen3 group, LVDP was lower at T2 and +dp/dtmax was lower at T4 and T5 (all P<0.05). In the I/R group, LVDP and +dp/dtmax at T2, T3, T4, and T5 were significantly different from those at T0 (all P<0.05). In the Fen3 group, +dp/dtmax decreased significantly at T4 and T5 (both P<0.05) and compared to the Fen1 and Fen2 groups, the Fen3 group had significantly lower LVDP and +dp/dtmax. –dp/dtmax, a left ventricular diastolic function indicator, was significantly lower in the I/R, Fen1, and Fen3 groups at T3, T4 and T5, and in the Fen2 group at T3, compared to that at T0 in the corresponding groups (all P<0.05). Compared to the S group, –dp/dtmax was decreased in the I/R and Fen3 groups at T2, T3 and T5, in the Fen1 group at T3 and T5, and in the Fen2 group at T3 (all P*<*0.05). Compared to the Fen3 group, the –dp/dtmax in the Fen2 and Fen1 groups was closer to that in the S group.

**Table:**
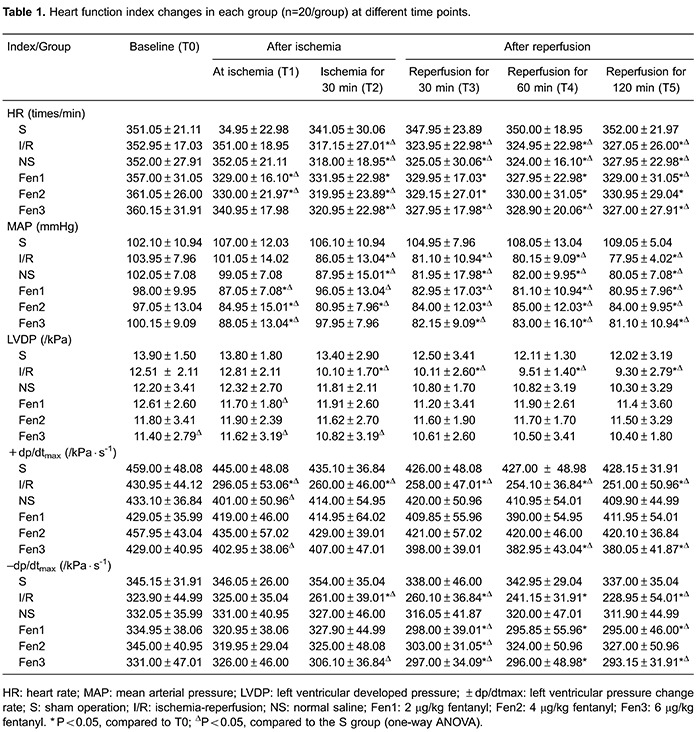

### Serum SOD, MDA, CK-MB, and cTnI concentrations

**Figure 1:**
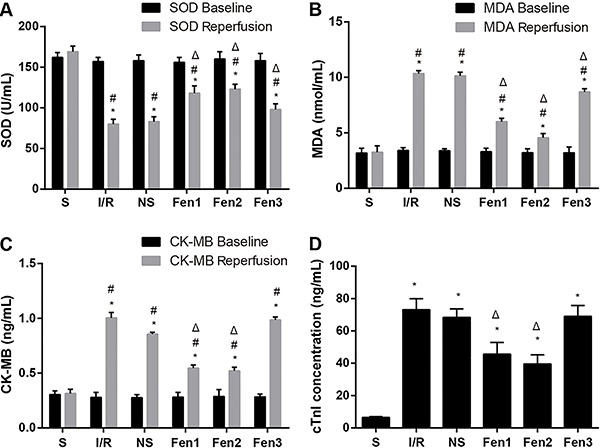
Superoxide dismutase (SOD) (*A*), malondialdehyde (MDA) (*B*), and creatine phosphokinase-MB (CK-MB) (*C*) in the sham operation (S) group, ischemia-reperfusion (I/R) group, normal saline (NS) group, and 2 μg/kg fentanyl (Fen1), 4 μg/kg fentanyl (Fen2), 6 μg/kg fentanyl (Fen3) groups at baseline and reperfusion for 120 min, and serum concentration of cardiac troponin-I (cTnl) (*D*) in each group. *P<0.05 comparing the SOD, MDA and CK-MB at baseline and reperfusion; ^#^P<0.05 compared to the S group; ^Δ^P<0.05 compared to the I/R group (one-way ANOVA).

There were no significant differences in SOD and MDA indicators between groups at T0 (all P*>*0.05). Except for the S group, the SOD value decreased, while the MDA increased in the other groups after reperfusion, compared to baseline values of SOD and MDA (all P<0.05). Compared to the S group, SOD values decreased, while MDA increased in the other five groups after reperfusion (all P<0.05). After reperfusion, MDA concentration was significantly decreased, while SOD activity was increased in the myocardial tissue of the Fen groups (all P<0.05) compared to the I/R group. However, there were no differences in the SOD value and MDA concentrations between the Fen groups, or between the NS and the I/R groups (all P*>*0.05; Figure 1A and B).

Serum CK-MB was similar at baseline (all groups, P*>*0.05). In addition to the S group, CK-MB values in the other groups were increased compared to the baseline value of CK-MB after reperfusion (all P*<*0.05). CK-MB values in the other groups were increased compared to the S group after reperfusion (all P<0.05). CK-MB values were lower in Fen1 and Fen2 groups after reperfusion (both P*<*0.05) compared to the I/R group. CK-MB values showed no significant differences between Fen3, NS and I/R groups after reperfusion (all P*>*0.05; Figure 1C).

Serum cTnI concentrations were significantly increased in the I/R group, Fen groups, and NS group (all P<0.05) compared to the S group. cTnI concentrations were significantly lower in the Fen1 and Fen2 groups (P*<*0.05) compared to the I/R group. However, there were no significant differences in cTnI concentration between Fen3, NS and I/R groups (P*=*0.355; Figure 1D).

### Histological and morphological changes in myocardial tissues

**Figure 2:**
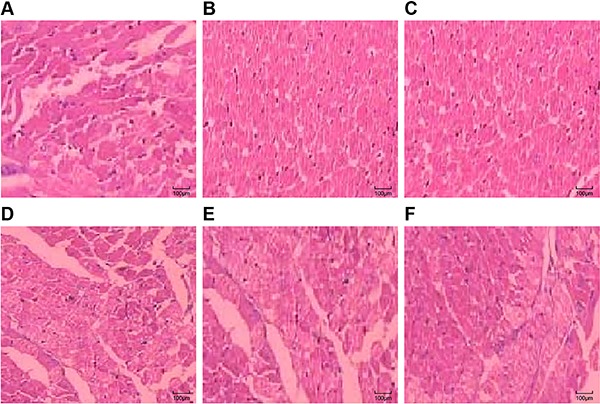
Hematoxylin-eosin staining of myocardial tissue in the sham operation (S) group (*A*), ischemia-reperfusion (I/R) group (*B*), normal saline (NS) group (*C*), fentanyl (Fen)1 group (2 μg/kg) (*D*), Fen2 group (4 μg/kg) (*E*), and Fen3 group (6 μg/kg) (*F*).

In the S group, myocardial tissue showed no significant pathological changes, cardiomyocytes presented elongated shapes, normal morphology, and parallel and neat arrangement to each other into a network shape, and thus myocardial cross striation can be observed; the cytoplasm and nucleus was uniform, the nuclear membrane was clear, and the nucleus was stained blue (Figure 2A). In the I/R group, myocardial pathological changes were obvious: parts of the myocardium presented regional lesions and necrosis; myocardium fiber arrangements were disordered and MI edges presented wavy shapes; the nucleus was condensed, fragmented and evenly dissolved; parts of the myocardium showed vacuolar degeneration and obvious myocardial interstitium edema was found; and a small amount of neutrophil infiltration and blood vessel necrosis, with leak bleeding (Figure 2B). In the NS and Fen3 groups, myocardial fibers were unevenly colored, disordered and slightly broken; the nucleus had condensation, rupture, dissolution, etc.; interstitial edema and a small amount of leak bleeding were found (Figure 2C and D). In Fen1 and Fen2 groups, myocardial cells had mild pathological changes, myocardial fibers were arranged neatly, and the cytoplasm was uniform; a small amount of fragmentation and dissolution, slight interstitial edema, and a small amount of leak bleeding were found (Figure 2E and F).

### Comparisons of MI area

**Figure 3:**
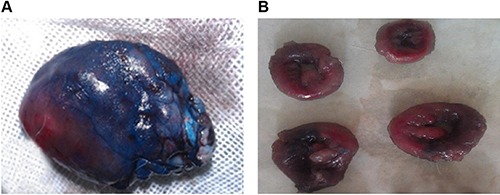
Evans Blue staining (*A*) and 2,3,5-triphenyl tetrazolium chloride (TTC) myocardium staining (*B*). Evans blue staining: blue color indicates normal myocardium and red color indicates ischemic myocardium; TTC myocardium staining: red color indicates ischemic myocardium and white color indicates myocardial infarction.

There were no significant differences in heart wet weight, LV or AAR/LV among the groups (all P>0.05). The MI area (IS/AAR%) was significantly reduced in the Fen1 and Fen2 groups compared to the I/R group (both P<0.05). There was no significant difference in the MI area between the Fen1 and Fen2 groups (P*=*0.721). The MI area showed a decreasing trend in the NS and Fen3 group compared to the I/R group with no significant differences, indicating that the protective effect of high concentrations of fentanyl was not significant on the heart (Table 2 and Figure 3).

**Table:**
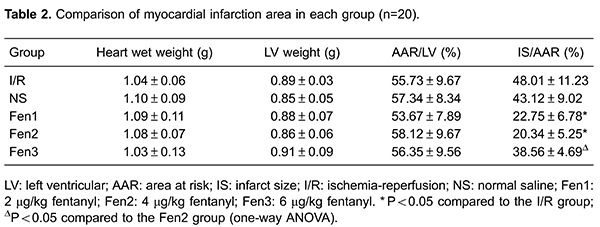

### Myocardial cell AI

**Figure 4:**
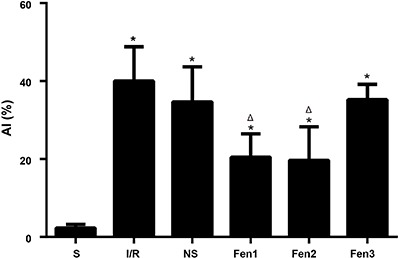
Comparison of the apoptosis index [AI (%)] in the sham operation (S) group, ischemia-reperfusion (I/R) group, normal saline (NS) group, and fentanyl (Fen)1 group (2 μg/kg), Fen2 group (4 μg/kg), and Fen3 group (6 μg/kg). *P<0.05 compared to the S group; ^Δ^P<0.05 compared to the I/R group (one-way ANOVA).

**Figure 5:**
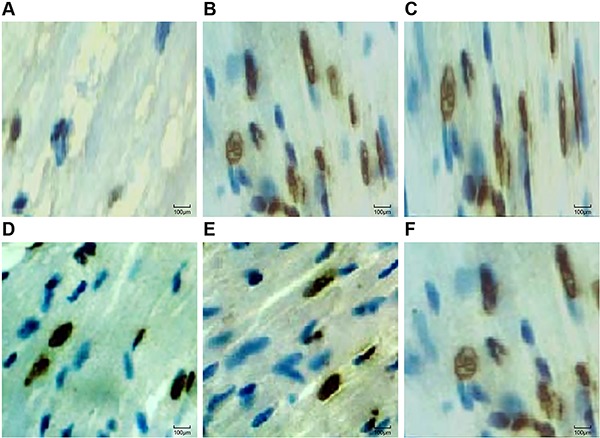
TUNEL staining detecting myocardial cells in the sham operation (S) group (*A*), ischemia-reperfusion (I/R) group (*B*), normal saline (NS) group (*C*), fentanyl (Fen)1 group (2 μg/kg) (*D*), Fen2 group (4 μg/kg) (*E*), and Fen3 group (6 μg/kg) (*F*). TUNEL: terminal deoxynucleotidyl transferase-mediated dUIP nick-end labeling.

Normal myocardial cell nuclei were stained blue under the microscope. Apoptotic cell nuclei presented in dark brown, the cytoplasm showed no color and the cell nuclei showed pyknosis. TUNEL-positive cells in the slices of each group could be seen in the light microscope. A small amount of the TUNEL-positive cells were distributed in the S group, but the apoptotic cell number significantly increased after reperfusion. The AI was significantly increased in the other five groups compared to the S group (all P*<*0.05). Apoptotic myocardial cell number was significantly increased in the I/R group compared to the S group (P<0.05) and was distributed in groups. The number of apoptotic myocardial cells in the Fen1 and Fen2 groups was significantly lower than in the I/R group while significantly higher than in the S group (all P*<*0.05). Apoptotic myocardial cells in the NS and Fen3 groups were slightly reduced compared to the I/R group, while they were significantly higher than in the Fen1 and Fen2 groups (all P<0.05; Figures 4 and 5).

### Bcl-2 and Bax protein expression levels

**Figure 6:**
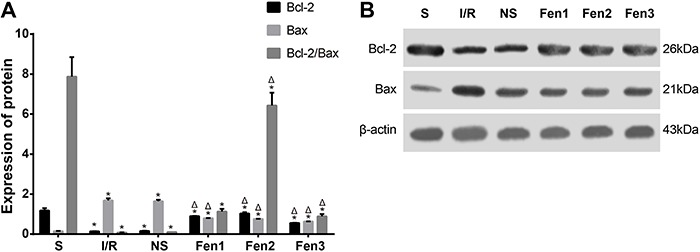
Bcl-2 and Bax protein expressions, and Bcl-2/Bax ratio in the sham operation (S) group, ischemia-reperfusion (I/R) group, normal saline (NS) group, and fentanyl (Fen)1 group (2 μg/kg), Fen2 group (4 μg/kg), and Fen3 group (6 μg/kg) (*A*, bar chart; *B*, electropherogram of western blot). Bcl-2: B-cell lymphoma 2; *P<0.05 compared to the S group; ^Δ^P<0.05 compared to the I/R group (one-way ANOVA).

Compared to the S group, Bax protein expression was significantly increased, Bcl-2 protein was significantly decreased, while Bcl-2/Bax protein ratio was decreased in the I/R group (all P*<*0.05). Bax protein was significantly decreased and Bcl-2 protein expression was significantly increased in the Fen1, Fen2 and Fen3 groups (all P<0.05) compared to the I/R group, while Bax and Bcl-2 protein expressions showed no significant differences between the three Fen groups (all P>0.05). The Bcl-2/Bax protein ratios were significantly increased in the Fen1, Fen2 and Fen3 groups (all P<0.05) compared to the I/R group, and the increase degree was significantly higher in the Fen2 group compared to that in the Fen1 and Fen3 groups. Bax and Bcl-2 protein expressions and Bcl-2/Bax protein ratio showed no significant differences between the NS group and the I/R group (all P>0.05; Figure 6).

### Bcl-2 and Bax mRNA expression levels

**Figure 7:**
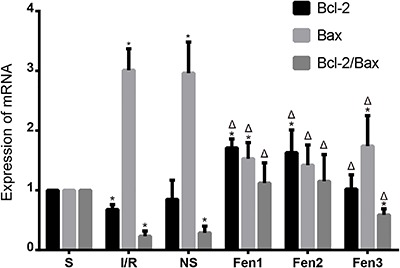
Bcl-2 and Bax mRNA expressions, and Bcl-2/Bax ratio in the sham operation (S) group, ischemia-reperfusion (I/R) group, normal saline (NS) group, and fentanyl (Fen)1 group (2 μg/kg), Fen2 group (4 μg/kg), and Fen3 group (6 μg/kg). Bcl-2: B-cell lymphoma 2. *P<0.05 compared to the S group; ^Δ^P<0.05 compared to I/R group (one-way ANOVA).

Bax mRNA expression levels were significantly increased in the I/R, NS and Fen groups (all P*<*0.05) compared to the S group. Bcl-2 mRNA expression levels were decreased in the I/R and NS groups and increased in the Fen1 and Fen2 groups (all P*<*0.05). Bcl-2/Bax mRNA expression ratios were significantly decreased in the I/R, NS and Fen3 groups compared to the S group (all P*<*0.05), while there were no significant differences in Bcl-2/Bax mRNA expression ratios between the Fen1, Fen2 and S groups (all P>0.05). Bax mRNA expression levels were significantly reduced compared to the I/R group, while Bcl-2 mRNA expression levels were significantly increased in the Fen1, Fen2, and Fen3 groups (all P<0.05). There were no significant differences in Bax and Bcl-2 mRNA expression levels between the Fen groups. Bcl-2/Bax mRNA expression ratios were significantly increased in the Fen1, Fen2, and Fen3 groups compared to the I/R group, while Bcl-2/Bax mRNA expression ratios were significantly higher in the Fen1 and Fen2 groups than those in the Fen3 group (all P<0.05; Figure 7).

## Discussion

This study assessed the effect of different concentrations of fentanyl preconditioning on cardiomyocyte apoptosis induced by I/R in rats. We established an I/R rat model for this study and our heart function results showed that, compared to the S group, HR and MAP were all decreased in the other groups at different time points except at baseline time. LVDP and ±dp/dtmax were lower in the I/R process of the I/R group, which indicated that the I/R process can affect heart function, and thus our model was successfully established. It was also found that LVDP and +dp/dtmax were lower in the Fen3 group compared to those in the Fen1 and Fen2 groups, and –dp/dtmax were closer in the Fen2 and Fen1 groups than that in the S group, which indicated that fentanyl may alleviate the heart dysfunction caused by I/R injury and that low and middle concentration had better effects. Fentanyl, as a potent and synthetic opioid analgesic, has a rapid onset and short duration of action, and it has been used to treat breakthrough pain (11,12). The opioid system has been reported to play various important roles in maintaining cardiac function by influencing cardiac rhythm and even developmental processes (20). It has been reported that fentanyl could protect against infarction by mediating both delta-opioid receptors and protein kinase C (21).

In order to study the mechanism of fentanyl on heart protection, other related indexes were measured. One of our results showed that compared to the I/R group, MDA concentration was significantly decreased, while SOD activity was increased in the myocardial tissue of the Fen groups after reperfusion, indicating an antioxidant role of fentanyl in myocardial cells during the I/R processes. MDA can damage biofilm by lipid peroxidation, while SOD can reduce lipid peroxidation by regulating the balance of body oxidation (22). A decreased SOD activity cannot eliminate the excessive oxygen-free radicals, which results in the formation of a large amount of MDA (23). We also found that compared to the I/R group, CK-MB values and cTnI concentration were lower in the Fen1 and Fen2 groups after reperfusion. As widely accepted, cTnI is highly expressed in cardiac muscle and also a preferred biomarker in the identification of MI (24). A prior study suggested that cTnI and CK-MB were released after heart surgery (25). On this ground, we believe that fentanyl may be useful in cushioning I/R injury. A clinical study also reported that the utilization of opioids aids in reducing the release of CK-MB and cTnI (26).

Our study also showed that, compared to I/R group, the MI area was significantly reduced and apoptotic myocardial cells were lower in the Fen1 and Fen2 groups, indicating that low and middle doses of fentanyl can protect cardiomyocyte apoptosis induced by I/R. Sufentanil, an analogue of fentanyl, could limit MI size and protect the heart in a dose-dependent manner, and is mediated by the preservation of phosphorylation of connexin 43 (27). Our research revealed that fentanyl down-regulated protein and mRNA expressions of Bax and up-regulated protein and mRNA expressions of Bcl-2 to resist I/R injury and increased Bcl-2/Bax ratios. In vertebrates, apoptosis mostly occurs via regulation of Bcl-2, which involves alterations in the integrity of outer mitochondrial membrane (OMM) (28,29). Bax, one of the pro-apoptotic effector proteins of Bcl-2 that disrupts OMM, is known to cause mitochondrial outer membrane permeabilization, causing the activation of caspases and cysteine proteases that initiate the cell destruction (30,31). In other words, fentanyl decreases apoptosis by increasing Bcl-2 expressions and decreasing Bax expressions to protect from I/R injury.

Based on our findings, we propose that ischemic preconditioning with fentanyl has a cardioprotective effect in the prevention of reperfusion injury. Our study is in accordance with that of Rentoukas et al. (32) that suggested that the utilization of RIPC and morphine during primary percutaneous coronary intervention could reduce reperfusion injury. Although fentanyl was proven to be protective of I/R damage, the optimal dose may be a discussion-worthy issue. We found that the Fen 3 group, though presenting less I/R damage than the I/R group, had less protective effects than the Fen 1 and Fen 2 groups. From this result, we speculate that the accumulating fentanyl may stimulate an overwhelming amount of opioid receptors and, therefore, leads to antagonism. However, our study is preliminary. To validate our findings, more clinical studies are needed.

In conclusion, fentanyl preconditioning may have antioxidant roles in myocardial cells during the I/R processes and may reduce the apoptosis of myocardial cells caused by I/R injury by down-regulating the expression of Bax, as well as up-regulating the expression of Bcl-2. Thus, fentanyl might be a potential medicine for I/R injury-related heart diseases.
